# ETHNIC IDENTIFICATION AND GRANDPARENT-GRANDCHILD RELATIONSHIPS IN ASIAN AND ASIAN AMERICAN YOUNG ADULTS

**DOI:** 10.1093/geroni/igac059.2341

**Published:** 2022-12-20

**Authors:** Catherine Ju, Brian Carpenter

**Affiliations:** University of Minnesota/Minneapolis VA, Eden Prairie, Minnesota, United States; Washington University in St. Louis, St. Louis, Missouri, United States

## Abstract

Asians and Asian Americans experienced an increase in exposure to racial discrimination during the COVID-19 pandemic. Ethnic minorities may counter discrimination by actively strengthening their ethnic identity and engaging in behaviors designed to enhance ethnic and cultural identification, such as reaching out to people who personify their culture. Grandparents are one such resource to whom young adults may turn to learn about their cultural heritage. The current study examined the degree to which facets of Asian and Asian American grandparent-grandchild relationships were related to ethnic identity, particularly in response to exposure to discrimination. Asian and Asian American young adults (N = 102) completed survey questions related to their experiences with COVID-19-related racial discrimination, ethnic identification, and relational closeness and frequency of contact with grandparents. Overall, exposure to discrimination was not significantly associated with strength of ethnic identity. However, there was a significant positive association between strength of ethnic identity and frequency of synchronous contact with grandparents, rs(100) = .329, p < .001. Strength of ethnic identity and relational closeness with grandparents were also significantly positively correlated, rs(100) = .383, p < .001. In contrast to some previous research, results show that discrimination encountered during the pandemic may not be related to strength of ethnic identification. However, there is a strong relationship between Asian and Asian American young adults’ strength of ethnic identification and the nature of their relationships with their grandparents. These findings enhance understanding of how intergenerational relationships are related to ethnic identity.

